# Few Layer Ti_3_C_2_ MXene-Based Label-Free Aptasensor for Ultrasensitive Determination of Chloramphenicol in Milk

**DOI:** 10.3390/molecules28166074

**Published:** 2023-08-15

**Authors:** Fang Li, Shuyue Xiong, Pei Zhao, Panpan Dong, Zijian Wu

**Affiliations:** Tianjin Key Laboratory of Food Biotechnology, College of Biotechnology and Food Science, Tianjin University of Commerce, Tianjin 300134, China; lifang_6952@163.com (F.L.); xiongshuyue9711@163.com (S.X.); zhaopei@tjcu.edu.cn (P.Z.); dongpp202202@163.com (P.D.)

**Keywords:** label-free, electrochemical aptasensor, chloramphenicol, Ti_3_C_2_ MXene nanosheets

## Abstract

Quantitative detection of veterinary drug residues in animal-derived food is of great significance. In this work, a simple and label-free electrochemical aptasensor for the highly sensitive detection of chloramphenicol (CAP) in milk was successfully developed based on a new biosensing method, where the single- or few-layer Ti_3_C_2_ MXene nanosheets functionalized via the specific aptamer by self-assembly were used as electrode modifiers for a glassy carbon electrode (aptamer/Ti_3_C_2_ MXene/GCE). Differential pulse voltammetry (DPV), electrochemical impedance spectroscopy (EIS), cyclic voltammetry (CV), scanning electron microscopy (SEM), atomic force microscope (AFM), and so on were utilized for electrochemical and morphological characterization. Under the optimized conditions, the constructed aptasensor exhibited excellent performance with a wider linearity to CAP in the range from 10 fM to 1 μM and a low detection limit of 1 fM. Aptamer/Ti_3_C_2_ MXene/GCE demonstrated remarkable selectivity over other potentially interfering antibiotics, as well as exceptional reproducibility and stability. In addition, the aptasensor was successfully applied to determine CAP in milk with acceptable recovery values of 96.13% to 108.15% and relative standard deviations below 9%. Therefore, the proposed electrochemical aptasensor is an excellent alternative for determining CAP in food samples.

## 1. Introduction

Animal-derived foods such as milk and eggs are an important part of daily diets. With the widespread use of veterinary drugs for the treatment and prevention of diseases in animals and the promotion of animal growth, the possible presence of veterinary drug residues in animal-derived foods is one of the key issues for food safety. Chloramphenicol (CAP), produced by Streptomyces Venezuelan [1], is an effective broad-spectrum antibiotic normally used in animals for treating and preventing infectious diseases [2]. However, excessive use of CAP can result in inevitable residues in animal-derived food, which subsequently bring about serious toxic side effects on human health through the food chain, such as aplastic anemia, gray baby syndrome, leukemia, nausea, diarrhea, and allergic reactions [3]. To date, various analytical methods, including high-performance liquid chromatography [4], liquid chromatography–mass spectrometry [5], and gas chromatography–mass spectrometry [6], have been developed for quantitively determining CAP in food samples. Although these methods are accurate and reliable, they have some inevitable limitations, such as their time-consuming nature, high cost, tedious operation procedures, professional operation skills, and excessive dependence on expensive and sophisticated instruments, all of which impede their application in the real-time monitoring of CAP. In addition, normally used microbial assays and enzyme-linked immuno-sorbent assays also have disadvantages [7,8], including low sensitivity, false positives, and cross-reactions. Otherwise, because they are simple, fast, and highly sensitive, various biosensing strategies for CAP detection have been put forward, such as surface plasmon resonance [9], colorimetric methods [10], fluoroimmunoassays [11], chemiluminescence [12], and electrochemical biosensors [13]. Among them, easy-operation electrochemical biosensors composed of a modifying electrode with functional nucleic acids (FNAs) have gained considerable interest for their better specificity and sensitivity [14].

Among these FNAs, aptamers (short-chain DNA or RNA with a specific sequence) are emerging as a novel class of nucleic-acid-based bio-recognition elements in developing biosensors or bioassays to detect various food contaminants [15], and when those aptamers on the transducer surface bind to a target, electrical or optical signals can be generated for quantification for measuring targets. When aptamers specifically bind to CAP after undergoing adaptive conformational changes [16], the electrochemical response changes, which is related to the concentration of CAP [17]. The aptamer-based electrochemical biosensors (electrochemical aptasensors) relying on the signal molecules labeled in the DNA/RNA strands have relatively better sensitivity. By the way, the existing labeling process makes the fabrication of aptasensors complicated and increases the cost [18,19]. It is expected that label-free aptasensors will be developed to enable sensitive determination of CAP.

In order to further improve the sensitivity of label-free aptasensors, some effective signal amplification strategies have been proposed, including DNA nanostructure self-assembly [20], hybridization chain reaction [21], exonuclease III- and DNAzyme-assisted methods [22], and so on. Especially, nanomaterials are used to enhance the sensitivity of aptasensors. MXene is a new type of metal carbide and metal nitride nanomaterial with a 2D-layered structure, and it has higher metallic conductivity, better biocompatibility, higher electrochemical performance, and a larger hydrophilic and chemical active surface area than traditional 2D materials. So it has been used to construct more sensitive biosensor devices. Feedstocks that are used to synthesize MXene are called MAX phases, and their general formula is M_n+1_AX_n_. The letter M represents an early transition metal, where A stands for an ⅢA or ⅣA element, X could be carbon or nitrogen, and n can be either 1, 2, or 3 [23]. MXenes are usually synthesized by liquid stripping techniques, such as ultrasonic stripping using hydrogen fluoride (HF), by which the A-layer (usually Al) in the MAX phase is selectively etched. Therefore, the chemical structure of MXene is finally expressed by the general formula M_n+1_X_n_Tx. Tx represents the terminal groups (-O, -F, and -OH) on the surface of MXene [24]. Ti_3_C_2_ is the most commonly utilized of MXene, which can be described as three layers of transition metal Ti covered with two layers of carbon, and a 2D-layered metal carbide with a similar structure to graphene. Since Ti_3_C_2_ MXene was successfully prepared in 2011, it has received a lot of attention focusing on electrochemical sensors [25], but there is limited research on aptasensors. The surface of Ti_3_C_2_ MXene is rich in oxygen or hydroxyl groups, which facilitate its interaction with other materials via hydrogen bonding, van der Waals interactions, electrostatic interactions, and coordination bonds. Thus, Ti_3_C_2_ MXene is considered a suitable matrix for constructing aptasensors. Hongyuan Cui et al. prepared an aptasensor based on Ti_3_C_2_ MXene for the detection of two diabetes biomarkers, with a low detection limit of 36 pM for insulin and 45 pM for vaspin [26]. Haiyan Wang et al. prepared cDNA-ferrocene/Ti_3_C_2_ MXene nanocomposites to construct an electrochemical aptasensor to detect the breast cancer marker Mucin1. The linear relationship was good in the range of 1 pM~10 μM, and the detection limit of Mucin1 was as low as 0.33 pM [27]. Fengling Yue et al. made an electrochemical aptasensor to detect aminoglycoside antibiotics in milk based on ordered mesoporous carbon @Ti_3_C_2_ MXene; the proposed aptasensor presented a wide linear range, and the detection limit was 3.51 nM [28]. Studies have shown that Ti_3_C_2_ MXene has a higher affinity for single-stranded DNA (ssDNA) than double-stranded DNA (dsDNA) due to the formation of Ti_3_C_2_ MXene-ion-ssDNA [29]. According to the molecular dynamic simulation results, Ti_3_C_2_ MXene has almost no hydrogen bond with DNA strands, and its interaction with DNA is probably realized through the ion bridge [30,31]. Moreover, there are also studies indicating that ssDNA may adsorb on the surface of Ti_3_C_2_ MXene nanosheets through π–π stacking between the aromatic nucleobases of DNA and graphite structure of MXene [32].

**Figure 1:**
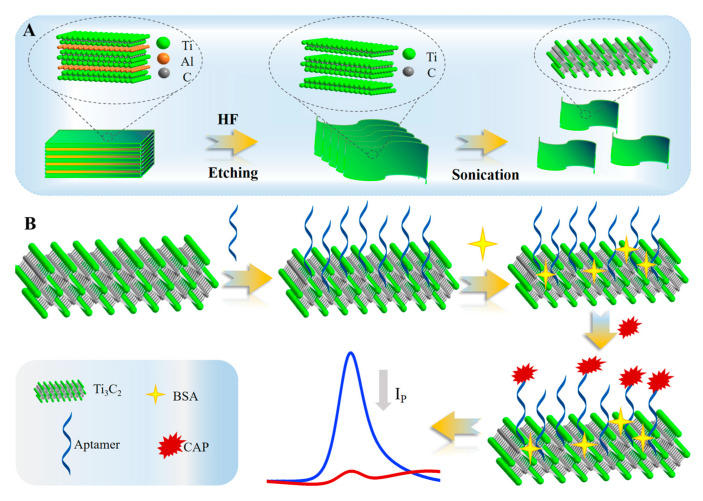
(**A**) The preparation of Ti_3_C_2_ MXene; (**B**) Schematic diagram of the fabrication of electrochemical aptasensor for CAP detection.

In this work, a simple Ti_3_C_2_ MXene-based electrochemical aptasensor with high sensitivity, selectivity, and stability for label-free detection of CAP in milk has been put forward, as shown in Figure 1. The stacking and aggregation of Ti_3_C_2_ MXene by van der Waals forces showed weaker electrochemical performance, and on the contrary, single- or fewer-layer Ti_3_C_2_ MXene nanosheets could provide more electron transport channels as well as more surface binding sites and a specific interface. When the aptamer specifically binds to CAP, electronic channels reduce, which results in a decrease in the response current related to the concentration of CAP. In addition, selectivity, reproducibility, and stability studies were carried out and obtained results that indicated that aptamer/Ti_3_C_2_ MXene/glassy carbon electrode (GCE) had shown an anti-interference property with long-term stability. The feasibility of the proposed aptasensor was verified by the analysis of real milk samples, which suggested that it was a new sensing platform for the detection of CAP in food samples.

## 2. Results and Discussion

### 2.1. Characterization of Ti_3_C_2_ MXene

**Figure 2:**
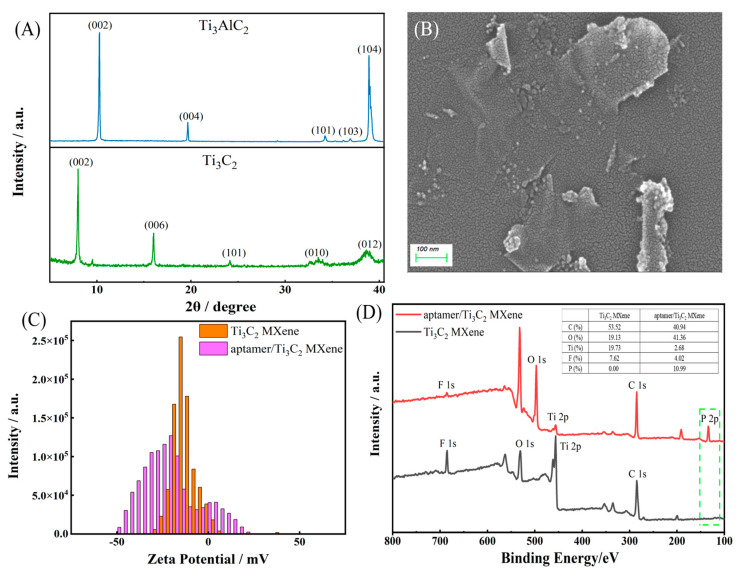
(**A**) XRD spectra of Ti_3_AlC_2_ and Ti_3_C_2_ MXene; (**B**) SEM image of Ti_3_C_2_ MXene; (**C**) zeta potential of Ti_3_C_2_ MXene and aptamer/Ti_3_C_2_ MXene; (**D**) wide XPS of Ti_3_C_2_ MXene and aptamer/Ti_3_C_2_ MXene.

The X-ray diffraction (XRD) features of Ti_3_AlC_2_ and Ti_3_C_2_ MXene are shown in Figure 2A. The (002) peak of Ti_3_C_2_ MXene shifted to a lower angle as compared to the commercial Ti_3_AlC_2_ phase, suggesting a successful removal of Al layers after HF exfoliation [33]. The appearance of the Ti_3_C_2_ MXene peak at approximately 25° was attributed to the (104) diffraction of TiO_2_, which demonstrated a certain amount of oxides. The morphology and structure of Ti_3_C_2_ MXene film were characterized by atomic force microscope (AFM) (Figure S1A,B) and scanning electron microscopy (SEM) (Figure 2D). Figure 2B,C shows the continuous coverage of Ti_3_C_2_ MXene on the electrode surface, that the thickness of films was less than 10 nm, and that the root mean square surface roughness was about 1.29 nm, corresponding to a single or few Ti_3_C_2_ MXene layers [34]. SEM images in Figure S2 showed a Ti_3_C_2_ cluster with the typical multilayer structure after HF exfoliation. Figure 2D revealed the typical morphology of a few layers of Ti_3_C_2_ MXene with a wrinkled paper-like surface, which could provide potential sites for effective ion and electron transport [35]. In order to explore the interaction between Ti_3_C_2_ MXene and aptamers, zeta potential was applied to investigate the charged states of Ti_3_C_2_ MXene and aptamer/Ti_3_C_2_ MXene as shown in Figure 2C, which indicated that they were all negatively charged and their combination may not be caused by electrostatic reaction [36]. To further validate the binding method between the aptamer and Ti_3_C_2_ MXene, X-ray photoelectron spectroscopy (XPS) was employed to analyze the elemental components and chemical states of both Ti_3_C_2_ MXene and the aptamer/Ti_3_C_2_ MXene. As presented in Figure 2D, the characteristic peaks of Ti 2p (455.8 eV), C 1s (284.8 eV), O 1s (530.5 eV), and F 1s (685.3 eV) elements appeared in the wide spectra of the Ti_3_C_2_ MXene and aptamer/Ti_3_C_2_ MXene. The appearance of P 2p (133.5 eV) and the increase in the percentage content of oxygen (19.13% to 41.36%) indicated the effective combination of Ti_3_C_2_ MXene and aptamer. The peak splitting spectrum of Ti, C, O, F, and P shown in Figure S3 further proved the effective synthesis of Ti_3_C_2_ MXene and the successful combination of Ti_3_C_2_ MXene and aptamer. Specifically, the Ti 2p XPS spectrum of Ti_3_C_2_ MXene was separated into Ti 2p_3/2_ and Ti 2p_1/2_ spin–orbit doublets, which showed five valence states ascribed to Ti (455.3 eV), Ti^2+^ (456.1/463.2 eV), Ti^3+^ (457.4 eV), Ti−C (461.6 eV), and TiO_2_ (459.4 eV). For the C 1s spectrum, the peak at 282.2 eV corresponded to C−Ti, whereas the peaks at 284.8, 286.5, and 288.9 eV corresponded to C−C, C−O, and C=O, respectively. The large component of C−C indicated the graphene−like nanostructure of the proposed Ti_3_C_2_ MXene nanosheets, maybe providing a strong π–π* stacking interaction with aptamer strands. Moreover, the O 1s spectrum showed three different oxygen bonds corresponding to O−Ti/O−H, O−C/O−H, and O−Ti, which were assigned to 529.9, 530.8, and 531.9 eV, respectively [37]. After being modified with an aptamer, the peak of Ti 2p spectra corresponding to the Ti^2+^ at 463.2 eV disappeared. Furthermore, the P 2p spectrum exhibited two peaks; the typical peak was attributed to the phosphate group (PO_4_^−^), and the other may be related to the chelation interaction between the PO_4_^−^ and Ti^2+^ [38].

### 2.2. Electrochemical Characterization of the Aptasensor Assembly Process

**Figure 3:**
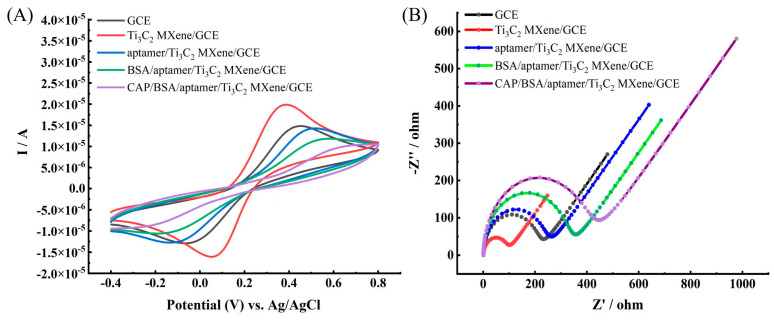
(**A**) CVs of bare GCE (black line), Ti_3_C_2_ MXene/GCE (red line), aptamer/Ti_3_C_2_ MXene/GCE (blue line), BSA/aptamer/Ti_3_C_2_ MXene/GCE (green line), and CAP/BSA/aptamer/Ti_3_C_2_ MXene/GCE (purple line); (**B**) EIS of bare GCE (black line), Ti_3_C_2_ MXene/GCE (red line), aptamer/Ti_3_C_2_ MXene/GCE (blue line), BSA/aptamer/Ti_3_C_2_ MXene/GCE (green line) and CAP/BSA/aptamer/Ti_3_C_2_ MXene/GCE (purple line). All the measurements were recorded in a solution containing 5 mM [Fe(CN)_6_]^3−^/^4−^ and 0.1 M KCl.

The change in the redox peak current (I_P_) in cyclic voltammetry (CV) is related to the electron transfer rate constant and thus the electron transfer resistance at different modification steps. The electrochemical measurements were performed in 5 mM [Fe(CN)_6_]^3−/4−^ at a scan rate of 0.1 V/s over a potential range of −0.4 V–0.8 V, respectively. CV for bare GCE, Ti_3_C_2_ MXene/GCE, aptamer/Ti_3_C_2_ MXene/GCE, Bovine serum albumin (BSA)/aptamer/Ti_3_C_2_ MXene/GCE, and CAP/BSA/aptamer/Ti_3_C_2_ MXene/GCE incubated electrodes are plotted in Figure 3. It can be seen from the black line and the red line that the current response of the electrode modified with Ti_3_C_2_ MXene was higher than that of the bare GCE. This was because of the high conductivity of Ti_3_C_2_ MXene, which could enhance the electron transfer rate. Subsequently, when Ti_3_C_2_ MXene/GCE was modified with an aptamer, it resulted in a decreased I_P_ (blue line) due to the negatively charged phosphate backbone in the aptamer that has a strong electrostatic repulsion on [Fe(CN)_6_]^3−/4−^ [39]. The excess active site was occupied with BSA to avoid nonspecific adsorption, and a continued decrease in the current was observed (green line), indicating that the modified BSA inhibited electron transfer to the electrode surface. The I_P_ decreased after the aptasensor was incubated with CAP (purple line), which could be ascribed to the formation of a complex, thereby obstructing electron transfer to the electrode surface.

Nyquist plots of impedance spectra for different modification steps at GCE provide detailed information on the charge transfer mechanisms in the modification process, as shown in Figure 3B. The diameter of the semicircle in the Nyquist plot determines the charge transfer resistance of the electrode surface, and larger semicircles are obtained when there are slow electron transfer kinetics. The bare GCE exhibited a large semicircle domain (black line), implying a very large electron transfer resistance. After the electrode was assembled with Ti_3_C_2_ MXene, the resistance significantly decreased (red line) as a result of improved conductivity by facilitating more electron transfer to the electrode interface. The immobilization of the aptamer induced an increase in resistance (blue line) due to the negatively charged phosphate backbone in the aptamer. This acted as an electrostatic barrier and repelled [Fe(CN)_6_]^3−/4−^ anions, which retarded the electron transfer to the electrode surface. Then, the resistance increased upon incubation with BSA (green line) and increased upon incubation with CAP (purple line) due to the reduced flow of electrons from the solution to the surface of the electrode. These electrochemical impedance measurement (EIS) results were complementary to CV, thereby indicating the successful fabrication of the aptasensor.

### 2.3. Optimization of Experimental Conditions

**Figure 4:**
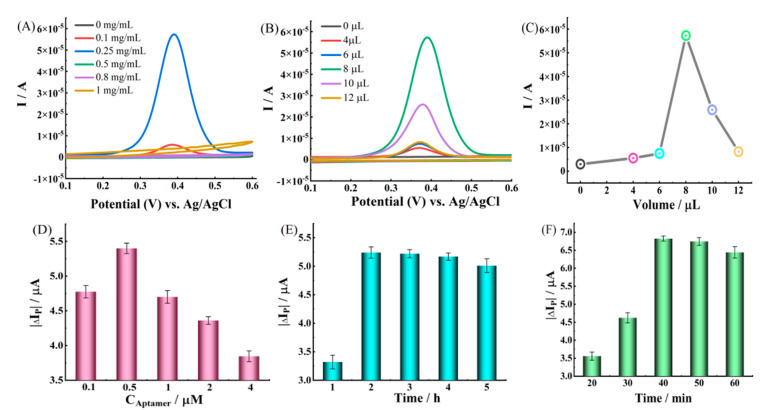
(**A**) the CVs for Ti_3_C_2_ MXene concentrations (0 mg/mL, 0.1 mg/mL, 0.25 mg/mL, 0.5 mg/mL, 0.8 mg/mL, and 1 mg/mL); (**B**,**C**) the CVs for volume of Ti_3_C_2_ MXene (0 μL, 4 μL, 6 μL, 8 μL, 10 μL, and 12 μL); (**D**) the effect of the concentration of aptamer on peak current of aptasensor, ΔI_P_ = I_P_, _aptamer/Ti_3_C_2_ MXene/GCE_−I_P_, _Ti_3_C_2_ MXene/GCE_; (**E**) the effect of the incubation time of aptamer on peak current of aptasensor, ΔI_P_ = I_P_, _aptamer/Ti_3_C_2_ MXene/GCE_−I_P_, _Ti_3_C_2_ MXene/GCE_; (**F**) the effect of the incubation time of CAP on peak current of aptasensor, ΔI_P_ = I_P_, _CAP/BSA/aptamer/Ti_3_C_2_ MXene/GCE_−I_P_, _BSA/aptamer/Ti_3_C_2_ MXene/GCE_.

To improve the analytical performance of the constructed electrochemical aptasensor, the experimental conditions, including the concentration and volume of Ti_3_C_2_ MXene, the concentration of aptamer, the incubation time of aptamer, and the incubation time of CAP, were optimized, as shown in Figure 4. As seen in Figure 4A, with the increase in the Ti_3_C_2_ MXene concentration in the range of 0–0.25 mg/mL, the I_P_ became higher, which was attributed to the fact that more electronic channels were provided to accelerate the transfer of electrons. However, when the concentration of Ti_3_C_2_ MXene was higher than 0.25 mg/mL, the excess Ti_3_C_2_ MXene would agglomerate on the electrode surface to form an electronic barrier layer, thus reducing the I_P_. Thus, 0.25 mg/mL Ti_3_C_2_ MXene was selected to fabricate the Ti_3_C_2_ MXene/GCE. The volume of Ti_3_C_2_ MXene was also investigated, shown in Figure 4B,C, in which the I_P_ increased with the increasing volume at the beginning (0 to 8 μL). The maximum current was obtained with 8 μL of Ti_3_C_2_ MXene suspension modified electrode, and the I_P_ decreased as the volume further increased. Similarly, this might be caused by the excess amount of Ti_3_C_2_ MXene possibly hindering the electron transfer. Hence, the aptasensor was prepared by 8 μL Ti_3_C_2_ MXene suspension modified on the GCE surface for further study.

As depicted in Figure 4D, the change in I_P_ initially increased with the increasing concentration of the aptamer. When the aptamer concentration reached 0.5 μM, the change in I_P_ reached its maximum value; subsequently, it gradually decreased. These observations suggest that the active sites on the surface of Ti_3_C_2_ MXene/GCE reach a state of complete saturation when the aptamer concentration reaches 0.5 μM. As the aptamer concentration continues to increase, over-saturation of the aptamer at the electrode surface will hinder electron transfer between the electrode surface and [Fe(CN)_6_]^3−/4−^ [39]. Therefore, a suitable concentration of 0.5 μM was selected for subsequent experiments. Immediately after, the influence of the aptamer incubation time on the aptasensor was studied, as shown in Figure 4E. The CAP aptamer was immobilized on the electrode surface and allowed to incubate for 1 h, 2 h, 3 h, 4 h, and 5 h, respectively. It was observed that the change in I_P_ reached its maximum at 2 h and subsequently stabilized, which indicated that the aptamer reached its saturation point at 2 h. Therefore, 2 h was considered the optimal incubation time for aptamer immobilization in subsequent experiments. The incubation time of CAP is also an important factor affecting aptasensor performance, which was optimized, shown in Figure 4F. The change in I_P_ reached its maximum value when the CAP incubation time was 40 min, and it was subsequently stabilized. These results suggest that a prolonged incubation time can provide a more sufficient reaction, bigger deviations of charge and resistance can be induce on an aptasensor, and then the increased I_P_ can be measured. Thus, the optimal incubation time for CAP in subsequent experiments was selected as 40 min.

### 2.4. Analytical Performance of the Electrochemical Aptasensors

**Figure 5:**
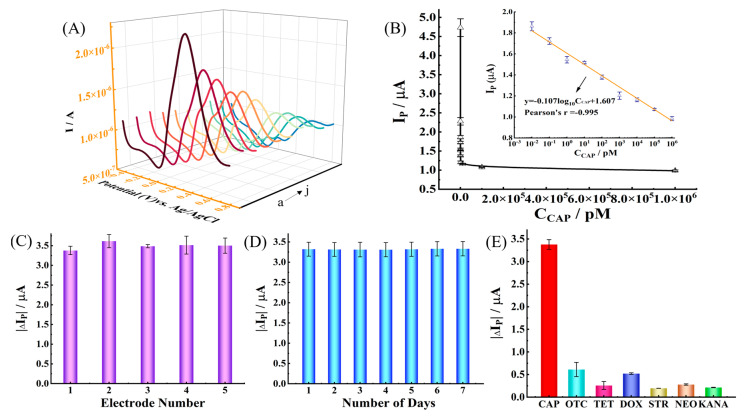
(**A**) DPV response of the aptasensor to different CAP concentrations (a to j respectively represents different concentrations of CAP: 1 fM, 10 fM, 100 fM, 1 pM, 10 pM, 100 pM, 1 nM, 10 nM, 100 nM, and 1 μM); (**B**) relationship between CAP concentration and DPV response. Inset: the alignment curve of the logarithm of CAP concentration and DPV response; (**C**) reproducibility of the aptamer/Ti_3_C_2_ MXene/GCE aptasensor for detection of 100 nM CAP, ΔI_P_ = I_P_, _CAP/BSA/aptamer/Ti_3_C_2_ MXene/GCE_−I_P_, _BSA/aptamer/Ti_3_C_2_ MXene/GCE_; (**D**) stability of the aptamer/Ti_3_C_2_ MXene/GCE aptasensor for detection of 100 nM CAP, ΔI_P_ = I_P_, _CAP/BSA/aptamer/Ti_3_C_2_ MXene/GCE_−I_P_, _BSA/aptamer/Ti_3_C_2_ MXene/GCE_; (**E**) selectivity of the constructed aptasensor. (the concentration of CAP, DOX, OTC, STR, NEO, KANA, and TET was 100 nM).

For the purpose of evaluating the performance of the prepared CAP aptasensor, the electrochemical responses of the sensors modified with different concentrations of CAP were measured by differential pulse voltammetry (DPV) under optimal conditions. In Figure 5A, it is noted that the I_P_ decreased as the concentration of CAP increased. A linear relationship was found between I_P_ and the concentration in the range of 10 fM–1 μM. The calibration curve is y = −0.107log_10_C_CAP_ + 1.607 (*Pearson’s r* = 0.995), and the limit of detection is 1 fM. In addition, the prepared aptasensor in this work was compared with previous reports, and the results are shown in Table 1. Compared with previous reports, the aptasensor has the merits of being simple, low-cost, a wider linear range, and having a lower detection limit.

The reproducibility of aptamer/Ti_3_C_2_ MXene/GCE was evaluated by DPV analysis of five identically prepared electrodes (Figure 5C). The relative standard deviation (RSD, *n* = 3) was 4.19% for CAP, showing that the aptamer/Ti_3_C_2_ MXene/GCE had very good reproducibility. The stability of the sensor was also tested by measuring the current responses for a week (Figure 5D). For 100 nM CAP, the RSD was 5.26%, and the I_P_ for CAP was reduced by 1.38% by the end of one week, depicting their acceptable stability. Generally, the aptasensor maintained its sensitivity for a week without significantly losing its current response. In order to evaluate the specificity of the developed electrochemical aptasensor, oxytetracycline (OTC), tetracycline (TET), doxycycline hydrochloride (DOX), streptomycin (STR), neomycin (NEO), and kanamycin (KANA) were used as interference substances. The effects of them on the current responses were assessed by DPV, as shown in Figure 5E. The response of the aptasensor to interference substances was significantly lower than CAP, indicating that the electrochemical responses were unaffected in the presence of potentially interfering antibiotics.

### 2.5. Detection of Chloramphenicol in Real Samples

In this experiment, the marked milk samples were taken as artificially contaminated milk and used for analysis of real samples so as to verify the feasibility of this method for detecting CAP in real food samples. The recovery rate of CAP was calculated based on the added value and the detected value in the same solution. Trace amounts of CAP standard solutions (10 pM, 10^3^ pM, 10^4^ pM, and 10^5^ pM) were added to the milk samples for detection. The detection data for the four samples are collected, respectively, in Table 2, with recovery rates ranging from 96.13% to 108.15% (*n* = 3), and the RSD varying from 2.637% to 8.342%. These results showed that the prepared electrochemical aptasensor based on Ti_3_C_2_ MXene can reliably detect CAP in milk.

## 3. Materials and Methods

### 3.1. Materials

The oligonucleotides of specific aptamer were synthesized by Sangon Biotechnology Co. Ltd. (Shanghai, China), which is 5′-ACTTC AGTGA GTTGT CCCAC GGTCG GCGAG TCGGT GGTAG-3′. The titanium aluminum carbide (Ti_3_AlC_2_, 95%) was purchased from Forsman Scientific Co., Ltd. (Beijing, China). CAP, DOX, OTC, STR, and KANA were purchased from Aladdin Biochemical Technology Co., Ltd. (Shanghai, China). NEO was purchased from Chaojiuba Biotechnology Co., Ltd. (Chengdu, China). TET was purchased from Yuanye Biotechnology Co., Ltd. (Shanghai, China). The HF was procured from Macklin. Phosphate buffered solution (PBS) (0.1 M, PH 7.4) was purchased from Yida Technology Co., Ltd. (Quanzhou, China). KCl was purchased from North Tianyi Chemical reagent Factory (Tianjin, China). Potassium ferrocyanide (K_4_[Fe(CN)_6_]·3H_2_O) was purchased from Kaitong Chemical Reagent Co., Ltd. (Tianjin, China). Potassium hexacyanoferrate (K_3_[Fe(CN)_6_]) was purchased from Bo Hua Chemical Reagent Co., Ltd. (Tianjin, China). Acetic acid (HAc, 36%) was purchased from Guangfu Technology Development Co., Ltd. (Tianjin, China). BSA was purchased from Aladdin Chemical Reagent Co., Ltd. (Beijing, China). Deionized water, distilled water, and ultra-pure (UP) water were used throughout the experiment.

### 3.2. Apparatus

All electrochemical measurements were conducted using the conventional three-electrode system, in which a GCE was employed as the working electrode (WE), a platinum wire was utilized as the counter electrode (CE), and a saturated Ag/AgCl electrode was used as the reference electrode (RE). Electrochemical experiments on CV and DPV were carried out with a CHI 660E electrochemical workstation (Beijing Huakoptian Technology Co., Ltd., Beijing, China). CV was recorded in the potential range of −0.2–0.8 V (vs. Ag/AgCl) and −0.2–0.6 V (vs. Ag/AgCl) in a 0.1 M PBS (PH 7.4) for the volume of Ti_3_C_2_ MXene and concentration of Ti_3_C_2_ MXene, respectively. Meanwhile, CV was recorded in the potential range of −0.4–0.8 V (vs. Ag/AgCl) in a 5 mM Fe(CN)_6_^3−/4−^ electrolyte solution containing 0.1 M KCl for optimizing the other experimental conditions. DPV was measured in the potential range of −0.4–0.8 V (vs. Ag/AgCl) in a 5 mM Fe(CN)_6_^3−/4−^ electrolyte solution containing 0.1 M KCl to determine the concentration of CAP.

The morphology of the prepared materials was characterized using SEM (S-3500 N, Hitachi, Tokyo, Japan) and AFM (Dimension Icon Bruker, Billerica, MA, USA), respectively. XPS was used for the element analysis, which was performed on an Axis Ultra DLD instrument (Kratos Analytical Ltd., Manchester, UK). XRD (Rigaku Co., Tokyo, Japan) was employed to analyze the structure of Ti_3_C_2_ MXene. The Zeta potential values of Ti_3_C_2_ MXene and aptamer/Ti_3_C_2_ MXene were investigated utilizing a Zetasizer nano-particle potentiometer (Tianjin Xina Intelligent Technology Co., Ltd., Tianjin, China).

### 3.3. Synthesis of Ti_3_C_2_ MXene

Ti_3_C_2_ MXene was synthesized utilizing Ti_3_AlC_2_ as the precursor by means of etching Al in HF solution (Figure 1A) [47]. The synthesis steps were as follows: First, 10.0 g of Ti_3_AlC_2_ powder was weighed and slowly added to 40% HF solution (100 mL) in a Teflon container. The mixture was stirred continuously at room temperature for 3 h and then diluted with deionized water (300 mL). The resulting diluted solution was centrifuged at 1467 RCF (relative centrifugal force) for 15 min. The sediment was collected and washed with deionized water until the PH reached 7.0. Then, the cleaned precipitate was vacuum dried at 100 °C for 6 h to obtain Ti_3_C_2_ MXene multilayer film. Afterward, 2.5 g of dry powder was weighed and added into distilled water (400 mL). After ultrasonic treatment for 2 h, the supernatant was centrifuged (367 RCF) for 15 min, and the supernatant was filtered by 0.22 mm filter membrane. After drying at 100 °C for 24 h, Ti_3_C_2_ MXene powder with single or fewer layers was obtained.

### 3.4. Construction of the Aptasensor

The procedure for making the aptasensor is illustrated in Figure 1B. Briefly, the GCE was polished with 0.3 μm and 50 nm alumina slurry until the surface of the electrode was specular sequentially, followed by thorough washing with ethanol solution and distilled water, respectively. Then, the prepared Ti_3_C_2_ MXene suspension (0.25 mg/mL, 8 μL) was dropped on the polished GCE (Ti_3_C_2_ MXene/GCE) and dried directly at room temperature. After rinsing with the deionized water, the modified electrode was incubated with aptamer solution (0.5 μM) for 2 h in air (aptamer/Ti_3_C_2_ MXene/GCE). Subsequently, the electrode was washed with deionized water to remove unbound aptamers. Due to the high affinity of Ti_3_C_2_ MXene towards ssDNA, it can facilitate the attachment of aptamer onto the electrode surface. Subsequently, the aptamer/Ti_3_C_2_ MXene/GCE was treated with 1% BSA (10 μL) to block non-specific binding sites and incubated at 4 °C for 1 h. After being thoroughly washed by deionized water several times, the prepared modified electrode (BSA/aptamer/Ti_3_C_2_ MXene/GCE) was finally stored at 4 °C for subsequent experiments.

### 3.5. Milk Sample Measurement

Real samples were purchased from a local supermarket (Tianjin, China) and analyzed for CAP. Firstly, 10 mL of UP water was used to dilute 10 mL of milk sample. Then, 20% HAc was added to precipitate the protein, and the diluted milk sample was adjusted to pH 4.6. Centrifuging (9168 RCF) for 25 min to remove the precipitated protein and retain the supernatant. Then, adjust the pH to 7.0. Therewith, the sample was filtered by 0.22 μm filter membrane [48]. Finally, different concentrations of CAP (10 pM, 1 nM, 10 nM, and 100 nM) were added to the milk samples and detected by the proposed electrochemical aptasensor. According to the detection results, the recovery rate was calculated, and the practicability of the sensor was evaluated.

## 4. Conclusions

In conclusion, a simple, environmentally friendly, highly selective, and sensitive electrochemical aptasensor was developed for determining CAP in milk. The excellent performance of the sensor is attributed to the significantly increased electroactive surface area and conductivity resulting from the extraordinary properties of the Ti_3_C_2_ MXene modifier. The proposed sensor manifests prime reproducibility, stability, and high selectivity with a detection range from 10 fM to 1 μM. The aptamer/Ti_3_C_2_ MXene/GCE showed an acceptable recovery rate of 96.13–108.15% with an RSD of less than 9% for the electrochemical determination of CAP in milk. Therefore, the proposed electrochemical aptasensor has a high potential for real-time monitoring of CAP in food samples.

## Figures and Tables

**Table 1:** Comparison with the other electrochemical methods for CAP detection.

Methods	Linear Range	LOD	Reference
PEI-rGO/AuNCs	5 pM~1 μM	2.08 pM	[40]
AgNPs/[NH_2_–Si]−f−GO	10 pM~0.2 μM	3.3 pM	[41]
Mn_2_O_3_@CCH	0.005~91.94 μM	0.03 μM	[42]
Fe_3_O_4_/N-rGO	1~200 μM	0.03 μM	[43]
MoS_2_-rGO	1~55 μM	0.6 μM	[44]
MSO NFs	0.003 μM~92.21 μM	1 nM	[45]
MXene-AuNP	0.0001~10 nM	0.03 pM	[46]
Ti_3_C_2_ MXene	10 fM~1 μM	1 fM	This work

Abbreviation: PEI-rGO/AuNCs: polyethyleneimine-functionalized reduced graphene oxide and gold nanocubes; AgNPs/[NH_2_–Si]−f−GO: graphene oxide and functionalized with (3−Aminopropyl) triethoxysilane/silver nanoparticles; Mn_2_O_3_@CCH: manganese oxide supported on carbon−modified halloysite nanotube; Fe_3_O_4_/N-rGO: Fe_3_O_4_/Nitrogen−doped reduced graphene oxide; MoS_2_-rGO: MoS_2_ modified reduced graphene oxide; MSO NFs: MnSnO_3_ NFs; MXene-AuNP: 2D transition of metal carbides loaded with gold nanoparticles.

**Table 2:** Detection results of CAP in milk.

Sample No.	Added (pM)	Found (pM)	Recovery (%)	RSD (%, *n* = 3)
1	10	10.314	103.14	2.637
2	1000	1058.791	105.88	8.342
3	10,000	10,815.439	108.15	6.953
4	100,000	96,126.457	96.13	7.736

## Data Availability

Not applicable.

## Supplementary Materials

The following supporting information can be downloaded at https://www.mdpi.com/article/10.3390/molecules28166074/s1, Figure S1: AFM 2D image and 3D image of Ti_3_C_2_ MXene; Figure S2: The SEM images of multilayered Ti_3_C_2_ MXene; Figure S3: XPS spectrum of Ti_3_C_2_ MXene and aptamer/Ti_3_C_2_ MXene: (A) The Ti 2p core spectra of Ti_3_C_2_ MXene; (B) The F 1s core spectra of Ti_3_C_2_ MXene; (C) The C 1s core spectra of Ti_3_C_2_ MXene; (D) The O 1s core spectra of Ti_3_C_2_ MXene; (E) The Ti 2p core spectra of aptamer/Ti_3_C_2_ MXene; (F) The P 2p core spectra of aptamer/Ti_3_C_2_ MXene.

## Sample Availability

Samples of the compounds are available from the authors.
