# Supplementary material for: Few Layer Ti3C2 MXene-Based Label-Free Aptasensor for Ultrasensitive Determination of Chloramphenicol in Milk
Source: Molecules. 2023 Aug 15;28(16):6074. doi: 10.3390/molecules28166074 (PMC10459553; doi:10.3390/molecules28166074)
Supplement: Supplementary file 1 [file molecules-28-06074-s001.zip › molecules-2529951-supplementary.pdf]

Few Layer Ti_3C_2 MXene Based Lable-free Aptasensor for Ultrasensitive Determination of Chloramphenicol in Milk

Fang Li , Shuyue Xiong , Pei Zhao , Panpan Dong and Zijian Wu *

Tianjin Key Laboratory of Food Biotechnology, College of Biotechnology and Food Science, Tianjin University of Commerce, Tianjin 300134, China

*Corresponding authors: E-mail: wzjian@tjcu.edu.cn

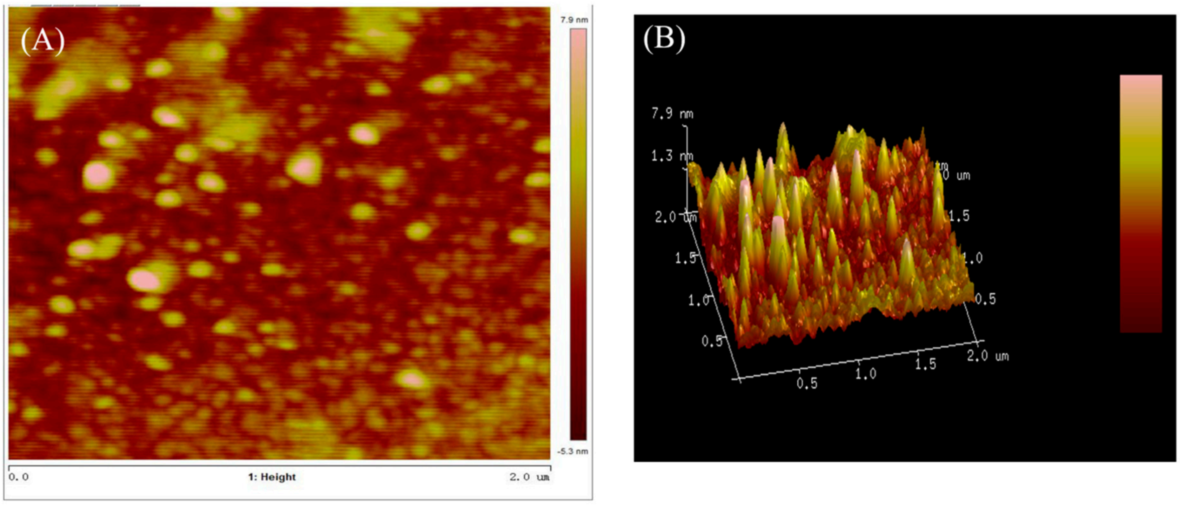

Figure S1. (A) AFM 2D image and (B) 3D image of Ti_3C_2 MXene.

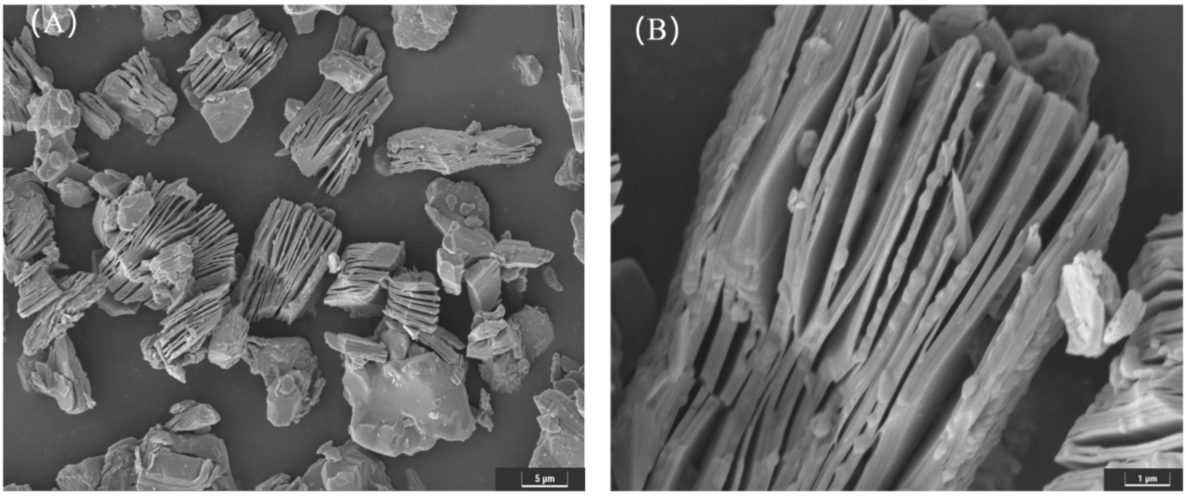

Figure S2. The SEM images of multilayered Ti_3C_2 MXene.

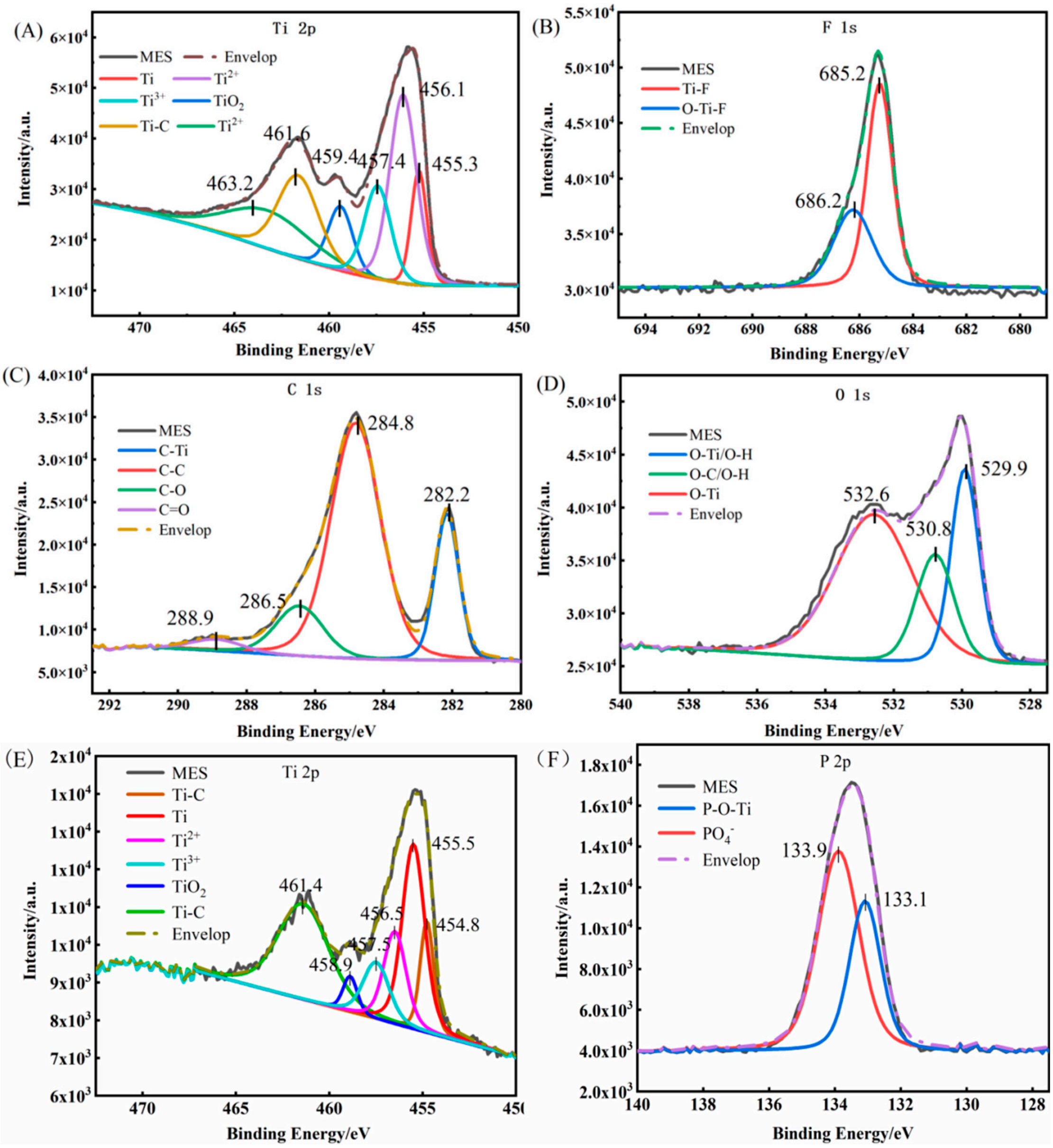

Figure S3. (A)The Ti 2p core spectra of Ti_3C_2 MXene; (B)The F 1s core spectra of Ti_3C_2 MXene; (C)The C 1s core spectra of Ti_3C_2 MXene; (D)The O 1s core spectra of Ti_3C_2 MXene; (E)The Ti 2p core spectra of aptamer/Ti_3C_2 MXene; (F)The P 2p core spectra of aptamer/Ti_3C_2 MXene.
